# Inhibition of angiogenesis and tumour growth by VEGF121–toxin conjugate: differential effect on proliferating endothelial cells

**DOI:** 10.1054/bjoc.2000.1439

**Published:** 2000-10

**Authors:** R Wild, M Dhanabal, T A Olson, S Ramakrishnan

**Affiliations:** SUGEN Inc, 230 East Grand Avenue, South San Francisco, CA, 94080–4811; Renal Division, Beth Israel Deaconess Medical Center and Harvard Medical School, Boston, MA, 02215; Department of Obstetrics and Gynecology, Duke University Medical Center, Durham, NC, 27710; Department of Pharmacology, University of Minnesota, 6–120 Jackson Hall, 321 Church St SE, Minneapolis, MN, 55455, USA

**Keywords:** angiogenesis, VEGF, vascular endothelial growth factor, KDR/flk-1, neuropilin-1, diphtheria toxin

## Abstract

Vascular endothelial growth factor (VEGF) plays an important role in tumour angiogenesis. VEGF binds to tyrosine kinase receptors, which are expressed almost exclusively on tumour endothelium. Therefore, VEGF can be used to target toxin molecules to tumour vessels for anti-angiogenic therapy. However, recent evidence suggests that VEGF can also bind in an isoform-specific fashion to a newly identified neuropilin-1 (NP-1) receptor. NP-1 is widely expressed in normal tissue and presents a potential target for unwanted toxicity. As a consequence, we investigated whether the VEGF121 isoform, which lacks the NP-1 binding domain, could be used to target toxin polypeptides to tumour vasculature. Treatment of endothelial cells with a VEGF121–diphtheria toxin (DT385) conjugate selectively inhibited proliferating endothelial cells, whereas confluent cultures were completely resistant to the construct. In addition, VEGF121–DT385 conjugate treatment completely prevented tumour cell induced angiogenesis in vivo. Most importantly, the conjugate inhibited tumour growth in athymic mice and induced tumour-specific vascular damage. There was also no apparent toxicity associated with the treatment. Our results suggest that proliferating endothelial cells are highly sensitive to VEGF121–toxin conjugates and that the binding to NP-1 receptors is not necessary for efficient inhibition of tumour growth. © 2000 Cancer Research Campaign


*British Journal of Cancer* (2000) **83**(8), 1077–1083
© 2000 Cancer Research Campaign
doi: 10.1054/ bjoc.2000.1439, available online at http://www.idealibrary.com on IDEAL®

### Inhibition of angiogenesis and tumour growth by VEGF121–toxin conjugate: differential effect on proliferating endothelial cells

**R Wild[1], M Dhanabal[2], TA Olson[3] and S Ramakrishnan[4]**

[1]SUGEN Inc, 230 East Grand Avenue, South San Francisco, CA 94080–4811; [2]Renal Division, Beth Israel Deaconess Medical Center and Harvard Medical School, Boston MA 02215; [3]Department of Obstetrics and Gynecology, Duke University Medical Center, Durham NC 27710; [4]Department of Pharmacology, University of Minnesota, 6–120 Jackson Hall, 321 Church St SE, Minneapolis MN 55455, USA

**Summary** Vascular endothelial growth factor (VEGF) plays an important role in tumour angiogenesis. VEGF binds to tyrosine kinase receptors, which are expressed almost exclusively on tumour endothelium. Therefore, VEGF can be used to target toxin molecules to tumour vessels for anti-angiogenic therapy. However, recent evidence suggests that VEGF can also bind in an isoform-specific fashion to a newly identified neuropilin-1 (NP-1) receptor. NP-1 is widely expressed in normal tissue and presents a potential target for unwanted toxicity. As a consequence, we investigated whether the VEGF121 isoform, which lacks the NP-1 binding domain, could be used to target toxin polypeptides to tumour vasculature. Treatment of endothelial cells with a VEGF121–diphtheria toxin (DT385) conjugate selectively inhibited proliferating endothelial cells, whereas confluent cultures were completely resistant to the construct. In addition, VEGF121–DT385 conjugate treatment completely prevented tumour cell induced angiogenesis in vivo. Most importantly, the conjugate inhibited tumour growth in athymic mice and induced tumour-specific vascular damage. There was also no apparent toxicity associated with the treatment. Our results suggest that proliferating endothelial cells are highly sensitive to VEGF121–toxin conjugates and that the binding to NP-1 receptors is not necessary for efficient inhibition of tumour growth. © 2000 Cancer Research Campaign

**Keywords:** angiogenesis; VEGF; vascular endothelial growth factor; KDR/flk-1; neuropilin-1; diphtheria toxin

The efficacy of conventional anti-tumour therapies is limited by several physical and physiological barriers (Jain, 1998). In addition, acquired resistance to chemotherapeutic drugs poses a major challenge to cancer therapy. Therefore, alternate strategies for cancer treatment are sought. Tumour growth beyond 1–2 mm in diameter requires the formation of new blood vessels from pre-existing vasculature, a process also called tumour angiogenesis (Folkman, 1992). Tumour angiogenesis is a complex process involving matrix degradation, endothelial cell (EC) proliferation, EC migration and tube formation. Positive and negative regulators control each step in angiogenesis. However, it is the eventual balancing act of these mediators that control the final angiogenic response (Iruela-Arispe and Dvorak, 1997).

Vascular endothelial growth factor (VEGF) has been considered as one of the most important positive regulators of tumour angiogenesis (Neufeld et al, 1999). VEGF belongs to the family of cystine knot proteins, which includes P1GF, VEGF-B, VEGF-C, VEGF-D and VEGF-E (Veikkola and Alitalo, 1999). VEGF is secreted as a disulphide-linked dimeric glycoprotein with five different isoforms (VEGF206, 189, 165, 145 and 121) being produced by means of alternate splicing (Neufeld et al, 1999). Three types of VEGF receptors have been identified, flt-1 (VEGFR-1), KDR/flk-1 (VEGFR-2) and flt-4 (VEGFR-3) (Neufeld et al, 1999). All three receptors consist of seven IgG-like domains, a transmembrane sequence and an intracellular kinase domain. VEGF binds preferentially VEGFR-1 and VEGFR-2, whereas VEGFR-3 interacts with VEGF-C and VEGF-D. More recently, an isoform-specific VEGF receptor was identified on endothelial and tumour cells, neuropilin-1 (NP-1), to which VEGF165 binds to via its exon 7 encoded heparin-binding domain (Soker et al, 1996; 1997). NP-1 has been previously associated with signaling axon guidance during neural development. However, new information shows that NP-1 enhances the mitogenic effects of VEGFR-2 signaling events in endothelial cells (Soker et al, 1998).

Receptors for VEGF (VEGFR-1 and VEGFR-2) with the exception of the newly identified NP-1, are expressed almost exclusively by endothelial cells and they are markedly over-expressed in the stromal vessels supplying tumours (Plate et al, 1993). Normal quiescent blood vessels show negligible levels of VEGFR-1 and VEGFR-2. Moreover, tumour-associated vascular endothelium proliferates at a faster rate than the normally quiescent endothelium (Baillie et al, 1995) and is therefore vulnerable to anti-proliferative endothelial cell-targeted therapy (Denekamp, 1982). As a consequence, selective destruction of tumour blood vessels results in histologically apparent thrombosis or haemorrhaged necrosis of the tumour (Burrows and Thorpe, 1993).

We have previously reported that a toxin polypeptide linked to the VEGF165 isoform can be used to target VEGF receptors and inhibit the proliferation of endothelial cells (Ramakrishnan et al, 1996). Furthermore, the VEGF165–toxin conjugate was able to inhibit angiogenesis in the chick chorioallantoic membrane assay. More importantly, VEGF165–toxin treatment leads to the inhibition of ovarian cancer growth in an experimental tumour model

*Received 27 January 2000*
*Revised 13 June 2000*
*Accepted 17 June 2000*

*Correspondence to:* S Ramakrishnan

(Olson et al, 1997). VEGF121 differs from all the other higher molecular weight species by the absence of the heparin-binding domain encoded by exon 7. VEGF121 binds preferentially to VEGFR-2 (Gitay-Goren et al, 1996) and does not bind to the NP-1 receptor, which is also expressed in adult heart and placenta. As a consequence, we were interested in testing whether the VEGF121 isoform could be used to prepare cytotoxic conjugates specific for endothelial cells. A truncated form of diphtheria toxin (DT385) was employed as the effector molecule. DT385 contains the catalytic domain and the translocation domain of diphtheria toxin but lacks the innate receptor-binding domain. DT385 was further genetically modified to incorporate a cysteine residue at the carboxyl terminus to facilitate chemical conjugation. DT385 catalytically ADP-ribosylates a unique histidine residue (diphthamide) of elongation factor-2, resulting in irreversible inhibition of protein synthesis. The present study shows that recombinant VEGF121 linked to DT385 efficiently inhibited endothelial cell proliferation and angiogenesis. More interestingly, the VEGF121–DT385 conjugate was selectively cytotoxic to proliferating endothelial cells and did not affect the viability of confluent cultures. Finally, the conjugate effectively inhibited the growth of tumours in vivo and specifically targeted tumour blood vessels without apparent toxicity. In summary, this study provides evidence that VEGF121-mediated toxin delivery differentially affects the proliferating endothelium. Moreover, we show that VEGF121–toxin conjugates can function as anti-angiogenic tumour therapies bypassing potential NP-1-mediated toxicity issues.

### MATERIALS AND METHODS

Culture conditions for HUVEC have been described (Ramakrishnan et al, 1996). Endothelial cells were used within the first six in vitro passages. The yeast expression system (*Pichia pastoris*) was purchased from Invitrogen (San Diego CA USA). The rat glioma cell line C6 (ATCC, Rockville MD, USA) and the human ovarian carcinoma cell line MA148 (University of Minnesota, USA) were maintained in RPMI-1640 medium (Life Technologies, Grand Island NY, USA) supplemented with 10% foetal bovine serum and antibiotics (penicillin and streptomycin, Life Technologies). Cultures were split twice a week at a ratio of 1:3.

### Expression and purification of VEGF121 and DT385

Cloning, expression and purification of VEGF121 have been previously described (Mohanraj et al, 1995). The diphtheria toxin polypeptide used in the present study was mutagenized by DNA-PCR to include a free cysteine residue at the carboxyl terminus of the truncated diphtheria toxin (DT385, residues 1–385 of the intact toxin molecule) to facilitate chemical conjugation. Expression and purification of the toxin polypeptide was performed as previously described (Mohanraj et al, 1996).

### Preparation and purification of VEGF121-toxin conjugate

Purified VEGF121 was chemically conjugated to DT385 with the heterobifunctional crosslinker *N*-succinimidyl-3-(2-pyridyldithio) propionate (SPDP; Pierce Chemical, Rockford IL, USA) as previously described (Ramakrishnan et al, 1996). VEGF121–toxin conjugate was subsequently purified using a Ni-NTA affinity column followed by size separation chromatography (Superdex-75; Amersham Pharmacia Biotech, Piscataway NJ, USA). A detailed description of the purification as been described previously (Ramakrishnan et al, 1996).

### Cytotoxicity assay

A detailed protocol on the cytotoxicity assay has been previously described (Ramakrishnan et al, 1996). For measuring inhibition of protein synthesis, VEGF–toxin treated cultures (48 h) were pre-incubated in leucine free medium for 30 min and then pulsed with 1 μCi of [$^3$H]leucine for 2 h. Radioactivity associated with the cells was determined after lysing the cells in 100 μl of 1.5 M sodium hydroxide. The radioactivity associated with control samples was considered as 100%. For measuring the sensitivity of proliferating vs quiescent HUVEC cultures, the VEGF121–toxin conjugate was added to cells on the 1st, 2nd, 3rd and 4th day after seeding. For each day, separate sets of control cultures were used. On day 4, control wells showed a 3-fold increase in [$^3$H]leucine incorporation when compared to day 1 control cultures.

### Chick chorioallantoic membrane (CAM) assay

A modified CAM assay was carried out to investigate tumour cell-induced angiogenesis (Gho et al, 1997). Here, exponentially growing MA148 cells were resuspended at $2 \times 10^7$ cells ml$^{-1}$ in serum-free medium, which contained 150 mg ml$^{-1}$ GELFOAM (absorbable gelatin powder; Upjohn, Kalamazoo MI, USA) in the absence or presence of VEGF121–DT385 toxin conjugate. 50 μl of the mixture was dispensed onto sterile plastic coverslips (Nalge Nunc International, Naperville IL, USA) and allowed to form a gel. The discs were then applied to the CAMs of 10-day-old embryos. After 72 h of incubation, CAMs were observed and photographed. Angiogenesis was assessed morphologically.

### Mouse matrigel assay

MA148 cells were resuspended at $2 \times 10^7$ cells ml$^{-1}$ in 10 mg ml$^{-1}$ Matrigel solution (Collaborative Biomedical Products; Becton Dickinson, Bedford MA, USA). 300 μl of the mixture was then injected subcutaneously into the right flank of 10-week-old female athymic nude mice (Harlan Sprague Dawley, Indianapolis IN, USA). Treatment was given daily by intraperitoneal injections of 30 μg VEGF121–DT385 conjugate or PBS in case of the control animals. Injections were administered for a 6-day period. Animals were sacrificed on day 7. Next, matrigels were removed surgically and subsequently observed for invasion of blood vessels into the matrix. The skin flaps associated with the matrigel were photographed and examined for blood-vessel density.

### Tumour growth inhibition studies

C6 rat glioma cells (ATCC, Rockville MD, USA) were resuspended at $2 \times 10^7$ cells ml$^{-1}$ in serum-free RPMI 1640 medium. 100 μl of the suspension was then injected subcutaneously into the flanks of 6–8-week-old athymic, nu/nu mice (Harlan Sprague Dawley). After allowing the tumour to establish (7 days), the animals were randomized and treatment was initiated. The mice were treated with daily intraperitoneal injections of 10 μg VEGF121–DT385 conjugate or equal amount of sterile saline for

© 2000 Cancer Research Campaign control animals for a period of 14 days. Tumour growth was monitored by caliper measurements. Tumour volume was calculated by the following formula:

$$\text{Tumour volume (mm}^3\text{)} = a \times b^2 \times \pi/6$$

where a = length (longer diameter) and b = width (shorter diameter).

Statistical significance between control and treated groups was determined by the Student's t-test. The experiment was repeated twice with at least six animals per group. Control tumours and residual tumours from treated animals were surgically removed at the end of the experiment and processed for immunohistochemical analysis.

The care and treatment of animals were in accordance with the University of Minnesota Institutional Animal Care and Use Committee (IACUC).

### Immunohistochemical studies

Tumour tissues were surgically removed and fixed with 10% buffered formalin (Sigma, St. Louis MO, USA). Paraffin embedded tissue sections were prepared and processed for immunohistochemical analysis. Sections were stained for endothelial cells with rabbit anti-human Factor VIII antibody (von Willebrand's factor; Dako Corp, Carpenteria CA, USA, 1:250 dilution). Sections were developed with ABC kit (Vecta, Burlingame CA, USA) and counterstained with haematoxylin (Sigma). Tumour-cell apoptosis was analysed by TUNEL assay (Boehringer Mannheim, Germany) and subsequently quantified using digital images captured with an OLYMPUS fluorescent microscope equipped with a CCD camera. The apoptotic index was estimated by counting the number of TUNEL-positive pixels per field using NIH Image analysis software (NIH, Bethesda MD, USA).

### RESULTS

The VEGF121–DT385 conjugate was purified by Nickel affinity and gel filtration chromatography. SDS-PAGE analysis was used to assess the purity of the conjugate (data not shown). We next tested the effect of conjugate treatment on endothelial cells. HUVEC cultures were treated with varying concentrations of the construct. After 48 h of incubation with VEGF121–DT385, cell viability was determined by measuring either protein or DNA synthesis. Cultures treated with the conjugate showed a progressive decrease in viability with increasing concentrations of conjugate. The addition of 100 nM conjugate almost completely inhibited protein synthesis in HUVECs (Figure 1A) and 50% inhibition was seen at 30 nM. DNA synthesis studies showed 50% inhibition at 50 nM concentration (data not shown). For comparative purpose a conjugate containing the VEGF165 isoform, which can interact with NP-1 receptors, was also used. Both conjugates showed very similar inhibition of protein synthesis (Figure 1A) as well as DNA synthesis (data not shown) in HUVEC. Whereas, equimolar concentrations of free DT385 showed no inhibitory effect (data not shown) illustrating the specific targeting of the toxin moiety via the VEGF isoforms. These results clearly demonstrate that targeting VEGFR-2 with a VEGF121–DT385 conjugate can efficiently inhibit protein synthesis and endothelial cell viability in vitro.

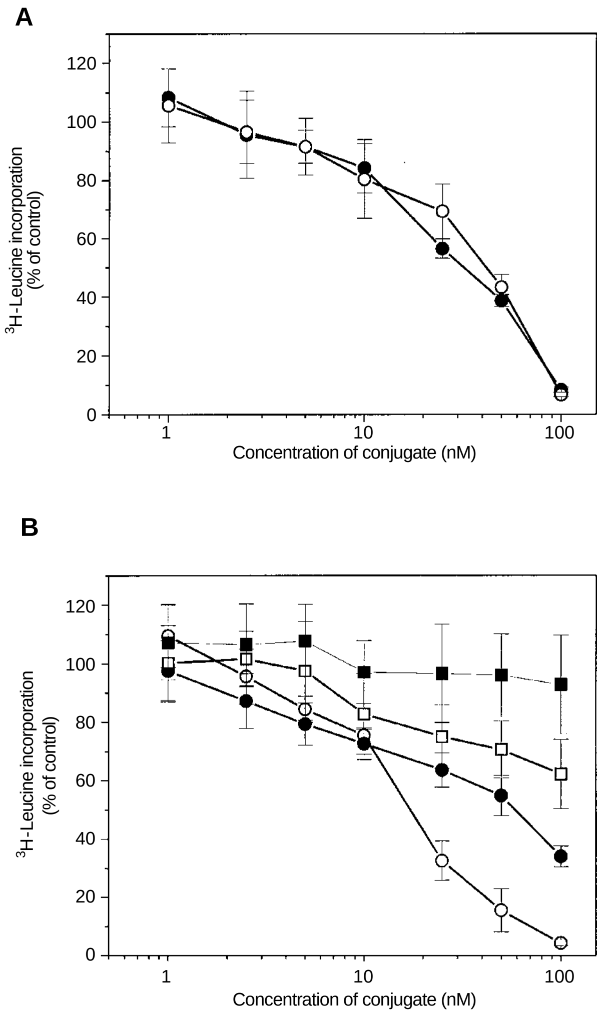

**Figure 1** (**A**) The cytotoxicity of VEGF121–DT385 toxin conjugate on human umbilical vein endothelial cells (HUVECs) was measured by [$^3$H]leucine incorporation. Each value is a mean of triplicate cultures from a representative experiment and error bars show standard deviation. ○ = VEGF165–DT385 conjugate. ● VEGF121–DT385 conjugate. Conjugate concentration 1 nM = 70 ng ml$^{-1}$. (**B**) The selective inhibition of protein synthesis was measured in proliferating vs confluent HUVEC cultures by the VEGF121–DT385 conjugate. Conjugate was added to HUVEC cultures on day 1, 2, 3 and 4 after initial seeding of cells. Two days later, cultures were pulsed with 1 μCi of [$^3$H]leucine. Each value is a mean of triplicate cultures from a representative experiment and error bars show standard deviation. ○ = Day 1; ● = Day 2; □ = Day 3; ■ = Day 4

To determine whether quiescent and proliferating endothelial cells are equally sensitive to conjugate treatment we performed the following experiment. Here, we treated HUVEC cultures on different days after initial seeding. The HUVEC culture doubling-time is approximately 18–24 h. Therefore, cultures become subconfluent by day 2, confluent by day 3 and turn into quiescent cultures by day 4 after seeding. Protein synthesis was used to assess the effect of the conjugate since DNA synthesis decreases as cultures become confluent. In contrast, protein synthesis in day 4 cultures was 3-fold higher when compared to day 1 control wells. Therefore, protein synthesis is a good indicator of cell viability in this type of experiment. Each time-point included its own control samples to which treatment groups were compared. Interestingly, only proliferating and subconfluent cultures were sensitive for VEGF121–DT385. As illustrated in Figure 1B,

© 2000 Cancer Research Campaign

100 nM conjugate inhibited [$^3$H]leucine incorporation in proliferating cells (day 1) by 95%. Similar concentrations of conjugate showed a progressively decreasing level of cytotoxicity when added to 2, 3 and 4-day old cultures. On day 2, when cultures were subconfluent, 100 nM VEGF121–DT385 conjugate inhibited protein synthesis by 66%. Three-day old confluent cultures were still less sensitive to the conjugate (38% inhibition) whereas 4-day old quiescent cultures were totally resistant to the construct. Reduced sensitivity of confluent cultures is not due to a decrease in protein synthesis. In fact, confluent cultures are highly active and showed approximately 3-fold higher levels of [$^3$H]leucine incorporation per well due to increased cell density (data not shown). Therefore, our results suggest that VEGF121–DT385 conjugate may also selectively inhibit proliferating endothelial cells in areas of active angiogenesis.

Two independent assay systems were employed to test the anti-angiogenic activity of the conjugate, the chick chorioallantoic membrane (CAM) assay and a nude mouse matrigel assay. In the CAM assay, MA148 cells were embedded in GELFOAM gelatin powder and loaded onto the CAMs of 10-day-old embryos. The incorporation of tumour cells into the gelatin matrix represented an effective angiogenic stimulus, as indicated by a visible increase in blood-vessel branch points. In contrast, control discs without tumour cells did not induce neovascularization (data not shown). When we included 1–3 μg VEGF121–DT385 toxin conjugate per disc, a potent inhibition of tumour cell-induced angiogenesis was recorded (Figure 2B). In fact, at the described concentration, a complete restraint of blood-vessel branching was observed. Free DT385 toxin at equimolar concentrations did not affect tumour cell-induced growth of blood vessels (data not shown) and results were comparable to PBS treated CAMs (Figure 2A). Established parent vessels were not affected by the conjugate, suggesting that normal tissue vasculature is not affected by the VEGF121–DT385 construct.

In the nude mouse matrigel assay, similar results were obtained with conjugate treatment. Tumour cells were incorporated into the matrigel matrix and the mixture was injected subcutaneously into the right flank of athymic nude mice. The matrigels were excised on day 7 and examined for blood vessels. PBS-treated animals showed extensive vascularization of the matrigel (Figure 2E). The skin flaps that were associated with the matrigel also displayed a high degree of blood-vessel density (Figure 2C). In contrast, VEGF121–DT385 conjugate treatment effectively prevented the invasion of blood vessels into the matrix. Excised matrigels of conjugate-treated mice appeared literally avascular (Figure 2F). Similarly, skin flaps associated with the matrigel revealed a clear inhibition of angiogenesis (Figure 2D). These experiments demonstrate that VEGF121 can effectively target toxin polypeptides to endothelial cells in vivo and that these toxin conjugates function as potent inhibitors of angiogenesis. VEGF121–DT385 toxin constructs did not affect the in vitro growth rate of the angiogenesis-inducing tumour cell line MA148 (data not shown). Therefore, the anti-angiogenic properties of the conjugate are due to its endothelial cell-specific action and not because of direct cytotoxic effects toward the tumour cells. Moreover, the phenomenon that established parent vessels were not affected by conjugate treatment supports the previous observation that quiescent endothelial cells are resistant to this construct.

Finally, VEGF–toxin conjugate was evaluated for its antitumour activity in a mouse model system. Here, athymic nude mice were transplanted with rat glioma cells (C6) and tumours were allowed

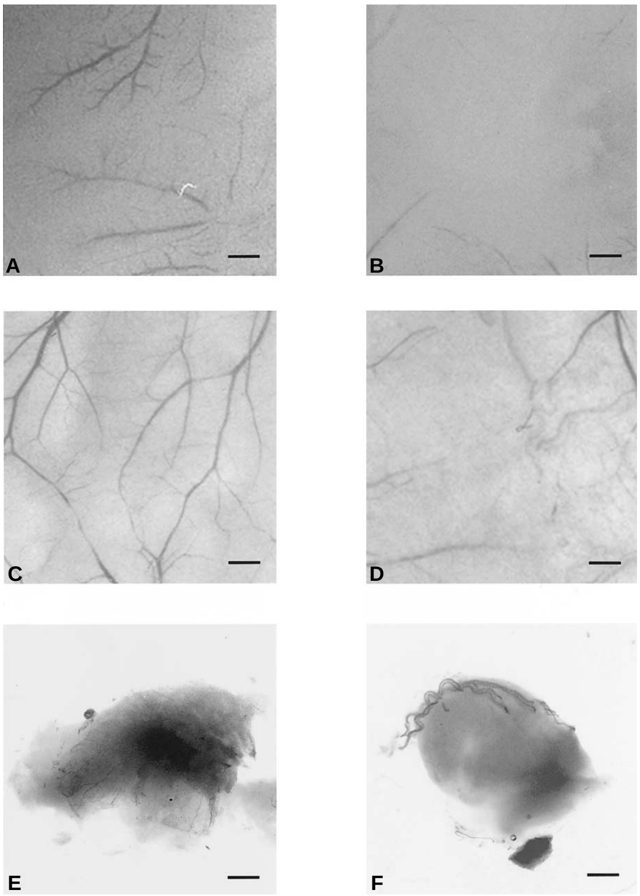

**Figure 2** Inhibition of tumour cell-induced angiogenesis by VEGF121–DT385. New blood-vessel growth was initiated by MA148, human epithelial ovarian carcinoma cells as described in 'Materials and methods'. Two independent angiogenesis assays (CAM assay and mouse matrigel assay) were used. (**A** and **B**) CAM assay, (**A**) control CAM (PBS-treated), (**B**) VEGF121–DT385 conjugate-treated CAM (3 μg disc$^{-1}$), (**C–F**) mouse matrigel assay, (**C**) skin flap of a control animal (PBS-treated), (**D**) representative skin flap of a VEGF121–DT385 conjugate-treated animal, (**E**) control (PBS) matrigel, (**F**) conjugate-treated matrigel (**A**–**D** scale bar = 1.5 mm; **E**, **F** scale bar = 2 mm)

to establish for 7 days. Considering that the maximum inhibitory activity in vitro was seen at 100 nM (7 μg ml$^{-1}$ conjugate) we estimated a daily dose of 10 μg VEGF121–DT385 conjugate per mouse for the in vivo study (the average blood volume of a 20 g mouse is approximately 1.6 ml). Conjugate was administered intraperitoneally every day for a period of 14 days. Tumour growth was monitored by caliper measurements. Data in Figure 3 show that conjugate treatment significantly inhibited the growth of tumours ($P < 0.01$). The smaller tumour size directly correlated with changes in the vasculature of the tumours in treated animals. A representative photomicrograph of Factor VIII staining, an endothelial cell-specific marker, is shown in Figure 4. A qualitative analysis revealed that compared to control tumours (Figure 4A), the conjugate-treated tumours had a lower number of vessels per field (Figure 4B). This result correlates well with a previous study, where VEGF165–DT385 conjugate treatment significantly reduced the microvessel density of a tumour as determined by computer assisted image analysis (Wild et al, 2000). Moreover, these studies revealed that conjugate treatment significantly reduces the vessel length, vessel number and branch points in

 *© 2000 Cancer Research Campaign*

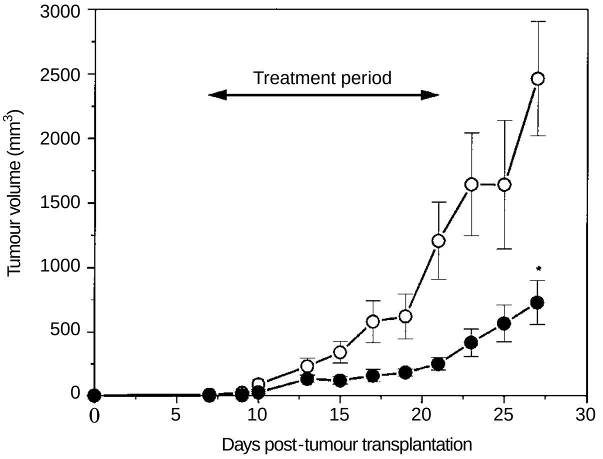

**Figure 3** Antitumour activity of VEGF121–DT385 conjugate. Female, athymic nude mice were transplanted with 2 × 10$^6$ C6 cells in a volume of 100 μl. After 7 days, mice were treated with conjugate (10 μg day$^{-1}$ intraperitoneally) for a 2-week period. Tumour growth was followed by caliper measurements. Each value is a mean of six animals with respective standard errors. ○ = PBS control; ● = VEGF121–DT385 conjugate-treated. The experiment was repeated with similar results; * shows statistical significance ($P$ < 0.01) as determined by Student's t-test

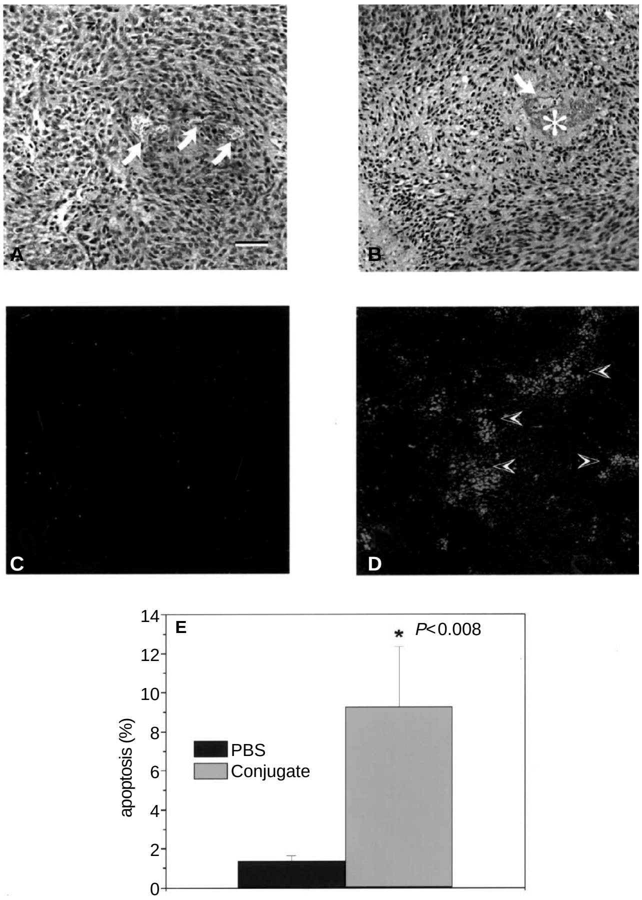

**Figure 4** Immunohistochemical analysis of tumour tissues. Paraffin embedded sections of tumour tissue were stained for von Willebrand's factor (factor VIII) and counterstained with haematoxylin. (**A** and **B**) Factor VIII staining, (**A**) PBS control, (**B**) VEGF121–DT385 conjugate-treated tissue. Arrows indicate factor VIII-positive vessels. * shows area of extravascular staining of factor VIII corresponding to a thrombotic lesion. Tumour tissue was also stained for TUNEL-positive cells to identify apoptotic activity, (**C–E**) TUNEL assay, (**C**) PBS control tumour tissue, (**D**) VEGF121–DT385 conjugate-treated tumour tissue. Arrows indicate location of tumour vessels, (**E**) apoptotic index as determined by counting the percentage of TUNEL-positive pixels per image. Scale bar = 50 μm tumour samples. Here, tumours from treated animals showed vascular damage and thrombotic lesions as revealed by the extravascular deposition of Factor VIII (Figure 4B). As a consequence, a large number of tumour cells, surrounding the damaged vessels, showed pyknotic nuclei and other characteristics of dead cells. Necrotic areas were evident up to 50–100 μm away from the damaged blood vessel, indicating that vascular targeting results in broad tumour cell death even in distant regions from the blood vessel.

This was further confirmed in TUNEL assays to detect apoptotic cells from these tumours. Control tumours showed very low numbers of TUNEL-positive cells (Figure 4C). In contrast, conjugate-treated tumours showed a large amount of apoptosis (Figure 4D). In fact, VEGF121–DT385 treatment resulted in an 8–9-fold increase in apoptotic index ($P$ < 0.008, Figure 4E). More interestingly, apoptotic cells were increasingly found in the vicinity of tumour vessels in conjugate-treated animals.

### DISCUSSION

Both bacterial and plant toxins have been used to prepare tumour cell-specific cytotoxic conjugates (Ramakrishnan et al, 1992). However, their delivery to solid tumours in vivo is limited due to a number of physical and physiological barriers (Jain, 1998). Therefore, targeting the tumour-associated vasculature instead of the tumour cells is advantageous. Tumour growth is dependent on the establishment of new blood vessels. Endothelial cells present in tumours exhibit many structural and biological differences when compared to normal tissues. For instance, endothelial cells in tumours proliferate actively (Denekamp, 1982; Baillie et al, 1995) and overexpress endoglin and VEGF receptors (Plate et al, 1993; Burrows et al, 1995). Consequently, toxin polypeptides can be targeted to these moieties by either the ligand or an antibody capable of binding to these receptors (Burrows & Thorpe, 1993; Ramakrishnan et al, 1996; Olson et al, 1997; Seon et al, 1997).

We previously showed that VEGF165–toxin conjugates efficiently inhibit tumour growth in vivo (Olson et al, 1997). VEGF165–toxin treatment resulted in vascular damage at the tumour without any apparent toxicity to normal tissues. Moreover, the antitumour activity of the conjugate was strictly associated with the anti-angiogenic properties of the construct, since the employed experimental tumour cell line did not express VEGF receptors. In contrast, a fusion toxin containing VEGF was found to be cytotoxic to Kaposi's sarcoma (KS) cell lines in vitro and in vivo (Arora et al, 1999). However, KS cells used in this report were positive for the VEGF receptors. Therefore, the observed antitumour activity is due to both direct and indirect effects of the fusion protein.

The biological activity of VEGF165 can be modulated by the heparin-binding domain, which is encoded by exon 7. Through this region, VEGF165 can also interact with NP-1. The VEGF121 isoform lacks this domain and therefore does not bind to NP-1. In

*© 2000 Cancer Research Campaign*

addition, VEGF121 binds selectively to VEGFR-2 compared to VEGFR-1. Both VEGFR-1 and VEGFR-2 have been found to be up-regulated at the tumour site (Plate and Risau, 1995). Therefore, targeting toxin polypeptides by VEGF165 could bind to VEGFR-1 and VEGFR-2 on tumour vessels as well as NP-1 on certain normal tissues. In contrast, VEGFR121 will selectively bind and deliver toxin molecules via VEGF-2 and bypasses toxin delivery to NP-1-positive cells. In this context, it was interesting to note that VEGF121–DT385 had a very similar cytotoxicity profile to HUVECs when compared to the VEGF165–DT385 conjugate. HUVEC cultures express VEGFR-1, VEGFR-2 as well as NP-1 (Soker et al, 1996; 1998). Furthermore, the administration of either the VEGF165–DT385 or the VEGF121–DT385 conjugate resulted in specific vascular damage at the tumour site. Normal vessels were not affected by the treatment (Olson et al, 1997). Therefore, it appears as if toxin delivery by VEGF molecules to endothelial cells is preferentially targeted via VEGFR-2. The presence or absence of exon 7 in the VEGF isoforms does not appear to change the overall anti-angiogenic activity of the conjugate. However, it may influence the toxicity profile of the construct. This is of particular interest, since NP-1 is highly expressed on neuronal cells. As a consequence, targeting toxins via VEGF165 may exert some underlying neurotoxic effects.

For the differential effect of conjugate on tumour vasculature, there are three possible explanations: proliferating endothelium, such as tumour-associated vessels, are more sensitive to the conjugate than normal, quiescent endothelium; VEGF receptors are highly expressed on tumour endothelium (Plate et al, 1993); VEGF receptor accessibility in normal vessels might be limited due to its abluminal distribution (Feng et al, 2000). However, at the tumour site, conjugates can escape from the circulation due to inherent leakiness of tumour vessels and bind to abluminal receptors. Our present studies provide evidence for the first hypothesis; VEGF121–DT385 conjugates selectively inhibited proliferating endothelial cells when compared to confluent cultures. It is possible that confluent cells have reduced numbers of VEGFRs. However, a similar phenomenon has been previously observed with an antibody–toxin conjugate against the transferrin receptor on human corneal endothelium (HCE). Both receptor number and affinity did not change between confluent vs proliferating cells (Fulcher et al, 1988). Alternatively, conjugate internalization and/or intracellular routing of the conjugate could be different in proliferating vs quiescent cells. Rapid delivery of the internalized toxin-constructs to lysosomes significantly reduces their cytotoxicity (Lappi, 1995). Therefore, differences in the functional status of the VEGF receptors may contribute to the differential sensitivity to the VEGF–toxin conjugate.

Lastly, we reported that inhibition of tumour angiogenesis by the VEGF121–DT385 conjugate resulted in the localized apoptotic death of tumour cells surrounding blood vessels. Although the current treatment protocol produced significant inhibition of tumour growth and temporary stabilization, it did not induce regression. Therefore, a prolonged treatment regimen may be desirable. Moreover, a recent report demonstrated that C6 gliomas undergo robust angiogenesis at the tumour periphery around week 4 of solid tumour development (Holash et al, 1999). Based on these results, a change in treatment schedule would be expected to significantly improve the antitumour activity of the conjugate. On the contrary, some tumour cells may still survive this vascular targeting strategy by coopting existing host vessels, which were not affected by the conjugate treatment. Under these circumstances, complementary approaches such as chemotherapy and radiation may have to be used in conjunction with anti-angiogenic therapies to accomplish complete eradication of tumours.

In summary, our results provide evidence that toxin delivery via the VEGF121 isoform can efficiently target endothelial cells, particularly proliferating or activated endothelial cells. In addition, VEGF121–toxin conjugates effectively inhibited tumour growth in vivo. The VEGF121 isoform might be a more suitable vascular targeting moiety since it does not bind NP-1 receptors, which are expressed in normal tissues. VEGF121 also preferentially binds VEGFR-2. However, our in vitro and in vivo data suggest, that both VEGF121 and VEGF165 might act in a similar mechanism on endothelial cells. Therefore, VEGF–toxin conjugates might preferentially target VEGFR-2 and do not require the binding to NP-1 receptors. Future experiments will focus on the potential difference in toxicity profile using the two different VEGF isoforms.

### ACKNOWLEDGEMENTS

This work was supported in part by a grant from NIH CA 71803 (SR), a grant from the Gustavus Louis Pfeiffer Research Foundation (SR) and a doctoral dissertation fellowship from the University of Minnesota (RW).

### REFERENCES

Arora N, Masood R, Zheng T, Cai J, Smith DL and Gill PS (1999) Vascular endothelial growth factor chimeric toxin is highly active against endothelial cells. *Cancer Res* **59**: 183–188

Baillie CT, Winslet MC and Bradley NJ (1995) Tumour vasculature–a potential therapeutic target. *Br J Cancer* **72**: 257–267

Burrows FJ and Thorpe PE (1993) Eradication of large solid tumours in mice with an immunotoxin directed against tumour vasculature. *Proc Natl Acad Sci USA* **90**: 8996–9000

Burrows FJ, Derbyshire EJ, Tazzari PL, Amlot P, Gazdar AF, King SW, Letarte M, Vitetta ES and Thorpe PE (1995) Up-regulation of endoglin on vascular endothelial cells in human solid tumours: implications for diagnosis and therapy. *Clin Cancer Res* **1**: 1623–1634

Denekamp J (1982) Endothelial cell proliferation as a novel approach to targeting tumour therapy. *Br J Cancer* **45**: 136–139

Feng D, Nagy JA, Brekken RA, Pettersson A, Manseau EJ, Pyne K, Mulligan R, Thorpe PE, Dvorak HF and Dvorak AM (2000) Ultrastructural localization of the vascular permeability factor/vascular endothelial growth factor (VPF/VEGF) receptor-2 (FLK-1, KDR) in normal mouse kidney and in the hyperpermeable vessels induced by VPF/VEGF-expressing tumours and adenoviral vectors. *J Histochem Cytochem* **48**: 545–556

Folkman J (1992) The role of angiogenesis in tumour growth. *Semin Cancer Biol* **3**: 65–71

Fulcher S, Lui GM, Houston LL, Ramakrishnan S, Burris T, Polansky J and Alvarado J (1988) Use of immunotoxin to inhibit proliferating human corneal endothelium. *Invest Ophthalmol Vis Sci* **29**: 755–759

Gho YS, Lee JE, Oh KS, Bae DG and Chae CB (1997) Development of antiangiogenin peptide using a phage-displayed peptide library. *Cancer Res* **57**: 3733–3740

Gitay-Goren H, Cohen T, Tessler S, Soker S, Gengrinovitch S, Rockwell P, Klagsbrun M, Levi BZ and Neufeld G (1996) Selective binding of VEGF121 to one of the three vascular endothelial growth factor receptors of vascular endothelial cells. *J Biol Chem* **271**: 5519–5523

Holash J, Maisonpierre PC, Compton D, Boland P, Alexander CR, Zagzag D, Yancopoulos GD and Wiegand SJ (1999) Vessel cooption, regression, and growth in tumours mediated by angiopoietins and VEGF. *Science* **284**: 1994–1998

Iruela-Arispe ML and Dvorak HF (1997) Angiogenesis: a dynamic balance of stimulators and inhibitors. *Thromb Haemost* **78**: 672–677

Jain RK (1998) Delivery of molecular and cellular medicine to solid tumours. *J Controlled Release* **53**: 49–67

*© 2000 Cancer Research Campaign*

Lappi DA (1995) Tumour targeting through fibroblast growth factor receptors. *Semin Cancer Biol* **6**: 279–288

Mohanraj D, Olson T and Ramakrishnan S (1995) A novel method to purify recombinant vascular endothelial growth factor (VEGF121) expressed in yeast. *Biochem Biophys Res Commun* **215**: 750–756

Mohanraj D, Wahlsten JL and Ramakrishnan S (1996) Expression and radiolabeling of recombinant proteins containing a phosphorylation motif. *Protein Expr Purif* **8**: 175–182

Neufeld G, Cohen T, Gengrinovitch S and Poltorak Z (1999) Vascular endothelial growth factor (VEGF) and its receptors. *Faseb J* **13**: 9–22

Olson TA, Mohanraj D, Roy S and Ramakrishnan S (1997) Targeting the tumour vasculature: inhibition of tumour growth by a vascular endothelial growth factor-toxin conjugate. *Int J Cancer* **73**: 865–870

Plate KH, Breier G, Millauer B, Ullrich A and Risau W (1993) Up-regulation of vascular endothelial growth factor and its cognate receptors in a rat glioma model of tumour angiogenesis. *Cancer Res* **53**: 5822–5827

Plate KH and Risau W (1995) Angiogenesis in malignant gliomas. *Glia* **15**: 339–347

Ramakrishnan S, Fryxell D, Mohanraj D, Olson M and Li BY (1992) Cytotoxic conjugates containing translational inhibitory proteins. *Annu Rev Pharmacol Toxicol* **32**: 579–621

Ramakrishnan S, Olson TA, Bautch VL and Mohanraj D (1996) Vascular endothelial growth factor–toxin conjugate specifically inhibits KDR/flk-1-positive endothelial cell proliferation *in vitro* and angiogenesis *in vivo*. *Cancer Res* **56**: 1324–1330

Seon BK, Matsuno F, Haruta Y, Kondo M and Barcos M (1997) Long-lasting complete inhibition of human solid tumours in SCID mice by targeting endothelial cells of tumour vasculature with antihuman endoglin immunotoxin. *Clin Cancer Res* **3**: 1031–1044

Soker S, Fidder H, Neufeld G and Klagsbrun M (1996) Characterization of novel vascular endothelial growth factor (VEGF) receptors on tumour cells that bind VEGF165 via its exon 7-encoded domain. *J Biol Chem* **271**: 5761–5767

Soker S, Gollamudi-Payne S, Fidder H, Charmahelli H and Klagsbrun M (1997) Inhibition of vascular endothelial growth factor (VEGF)-induced endothelial cell proliferation by a peptide corresponding to the exon 7-encoded domain of VEGF165. *J Biol Chem* **272**: 31582–31588

Soker S, Takashima S, Miao HQ, Neufeld G and Klagsbrun M (1998) Neuropilin-1 is expressed by endothelial and tumour cells as an isoform-specific receptor for vascular endothelial growth factor. *Cell* **92**: 735–745

Veikkola T and Alitalo K (1999) VEGFs, receptors and angiogenesis. *Semin Cancer Biol* **9**: 211–220

Wild R, Ramakrishnan S, Sedgewick J and Griffioen AW (2000) Quantitative assessment of angiogenesis and tumour vessel architecture by computer assisted digital image analysis: effects of VEGF–toxin conjugate on tumour microvessel density. *Microvasc Res* **59**: 368–376

*© 2000 Cancer Research Campaign*
